# Multiple Flaps for Trochanteric Pressure Sore Reconstruction: A Case Series

**DOI:** 10.7759/cureus.9369

**Published:** 2020-07-24

**Authors:** Luca Negosanti, Sara Tedeschi, Fabio F Trapani, Luca Gaiani, Rossella Sgarzani

**Affiliations:** 1 Specialized Care Unit, Montecatone Rehabilitation Institute, Imola, ITA; 2 Division of Infectious Diseases, S.Orsola-Malpighi University Hospital, Bologna, ITA; 3 Division of Orthopedics Surgery, Imola Hospital, Imola, ITA; 4 Plastic Surgery, Azienda Unità Sanitaria Locale Della Romagna, Cesena, ITA

**Keywords:** pressure sores, bone infection, flap reconstruction

## Abstract

Trochanteric pressure sores can be quite difficult to treat, especially in cases of large bone involvement requiring a wide debridement. The residual wound is large and deep, and the reconstruction must ensure a complete fill of all dead spaces, then must be covered with adequate tissue to allow for healing, and reduce the risk of recurrence.

We report a case series of spinal cord-injured patients affected by a trochanteric pressure sore. The reconstruction was achieved using a combination of muscle and a cutaneous muscle flap from the thigh. The result was complete healing of the wound with no recurrence at 18 months.

In these cases, muscle or musculocutaneous flaps are the better choices because they permit the use of a good volume of viable tissue. In some cases, the flap can be combined to obtain a better result.

## Introduction

Spinal cord injury (SCI) patients represent a population at high risk of pressure ulcer (PU); reported prevalence is highly variable and seems to be related to the level of spinal lesion. In a recent review of the literature, Cowan et al. reported a PU prevalence of 33.9% for patients with quadriplegia and 47.4% for patients with paraplegia [1]. Trochanteric pressure sores are always associated with osteomyelitis; therefore, the debridement must be wide to remove all infected or unviable soft and bony tissues. The golden standard is Gilderstone arthroplasty [2]. The residual wound is usually very large, deep, and the reconstruction must ensure a complete fill of all dead spaces and cover with adequate tissue to allow the healing and reduce the risk of recurrence. We report four cases of complex trochanteric wounds repaired by a combination of muscle and musculocutaneous flaps in spinal cord-injured patients.

## Case presentation

Four male patients affected by a SCI was referred to us for the treatment of a trochanteric pressure sore.

**Figure 1:**
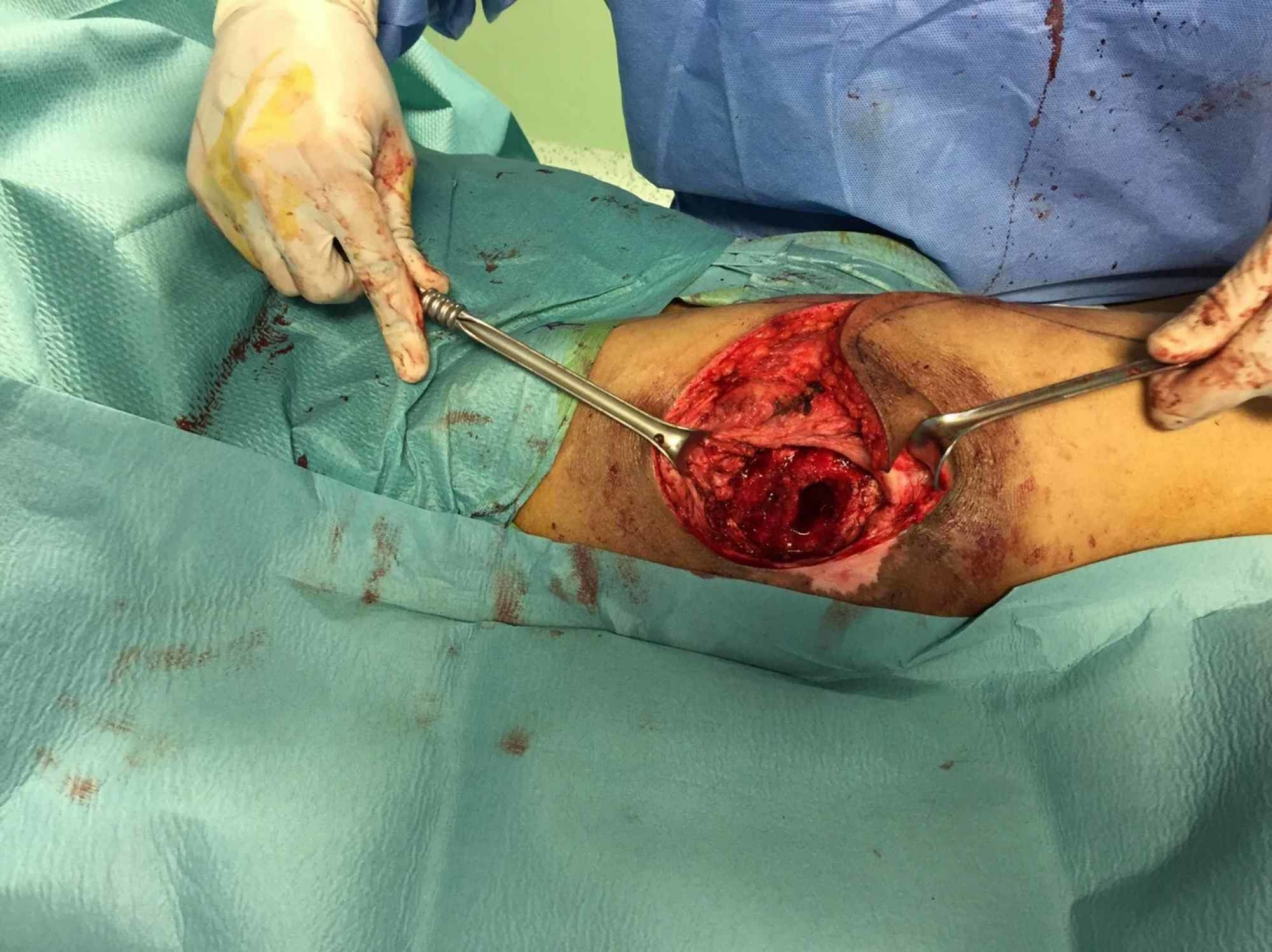
Right trochanter pressure sore.

All the wounds were characterized by purulent exudate and bone exposure. Imaging reported the presence of an inflammatory process on the great trochanter and the femural head. We planned for two surgical procedures. The first surgery consisted of a wide debridement of the wound. Through a horizontal incision, we exposed all the wound edges, and all macroscopically unviable soft tissues were removed. Bone excision involved the great trochanter and a part of the femural head that appeared unviable (Figure 1).

Specimens of soft and bone tissues were histologically and microbiologically analyzed to assess for the presence of infection. After hemostasis, the wound was treated with topical negative pressure therapy with polyurethane foam and negative pressure of 120 mmHg [3]. The initial empiric antibiotic therapy was piperacillin-tazobactam at 4.5 g, given four times each day; our protocol of empiric antimicrobial therapy is based on the revision of the most common micro-organisms in our intraoperative specimen cultures [4]. This antibiotic is expensive but covers a broad spectrum of micro-organisms and, in our series, we never needed to change this therapy once we received the results of intraoperative specimen cultures. This allowed us to administer an effective antimicrobial therapy from post-operative day 1. The analysis reported the presence of osteomyelitis in all the cases. After one month, we planned the reconstructive procedure. We decided to combine multiple flaps to obtain a complete fill of the cavity and adequate cover [5-8].

First patient was a 54-years-old male affected by paraplegia and a right trochanteric pressure sore; in this case, the reconstruction was achieved with a combination of rectus femoralis muscle and vastus lateralis musculocutaneous flaps. The second patient was a 52-years-old male affected by paraplegia and a right trochanteric pressure sore, while the third case was a 37-years-old male affected by paraplegia and a trochanteric pressure sore; the reconstruction, in these two cases, was achieved with rectus femoralis and vastus lateralis muscle flaps covered by a skin graft. The last patient was a 56-years-old male affected by paraplegia and a right trochanteric pressure sore; the reconstruction was achieved combining rectus femoralis, vastus lateralis, and gracilis muscles flap covered by a skin graft.

**Figure 2:**
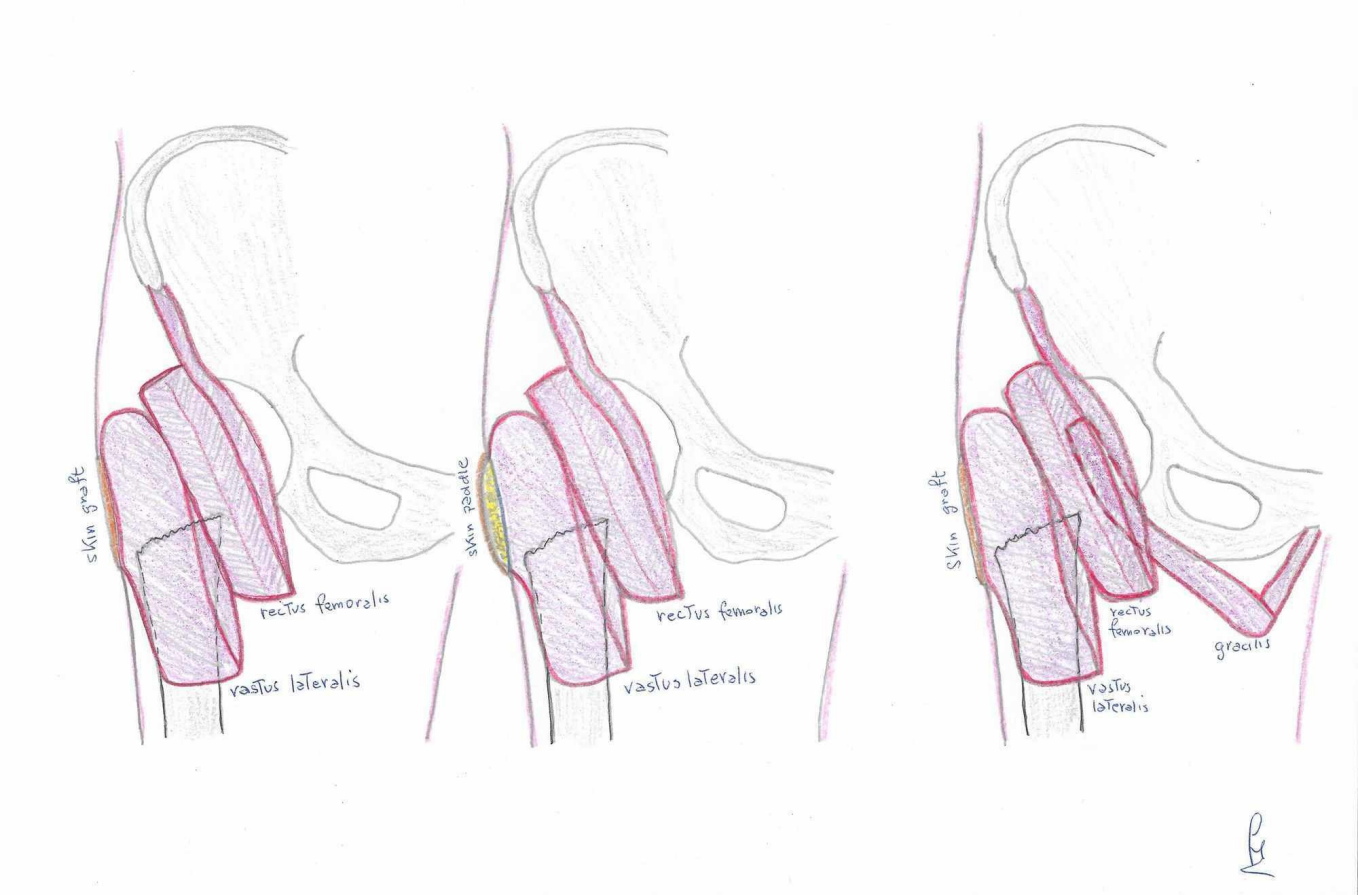
Planning of the reconstruction in the three different cases.

**Figure 3:**
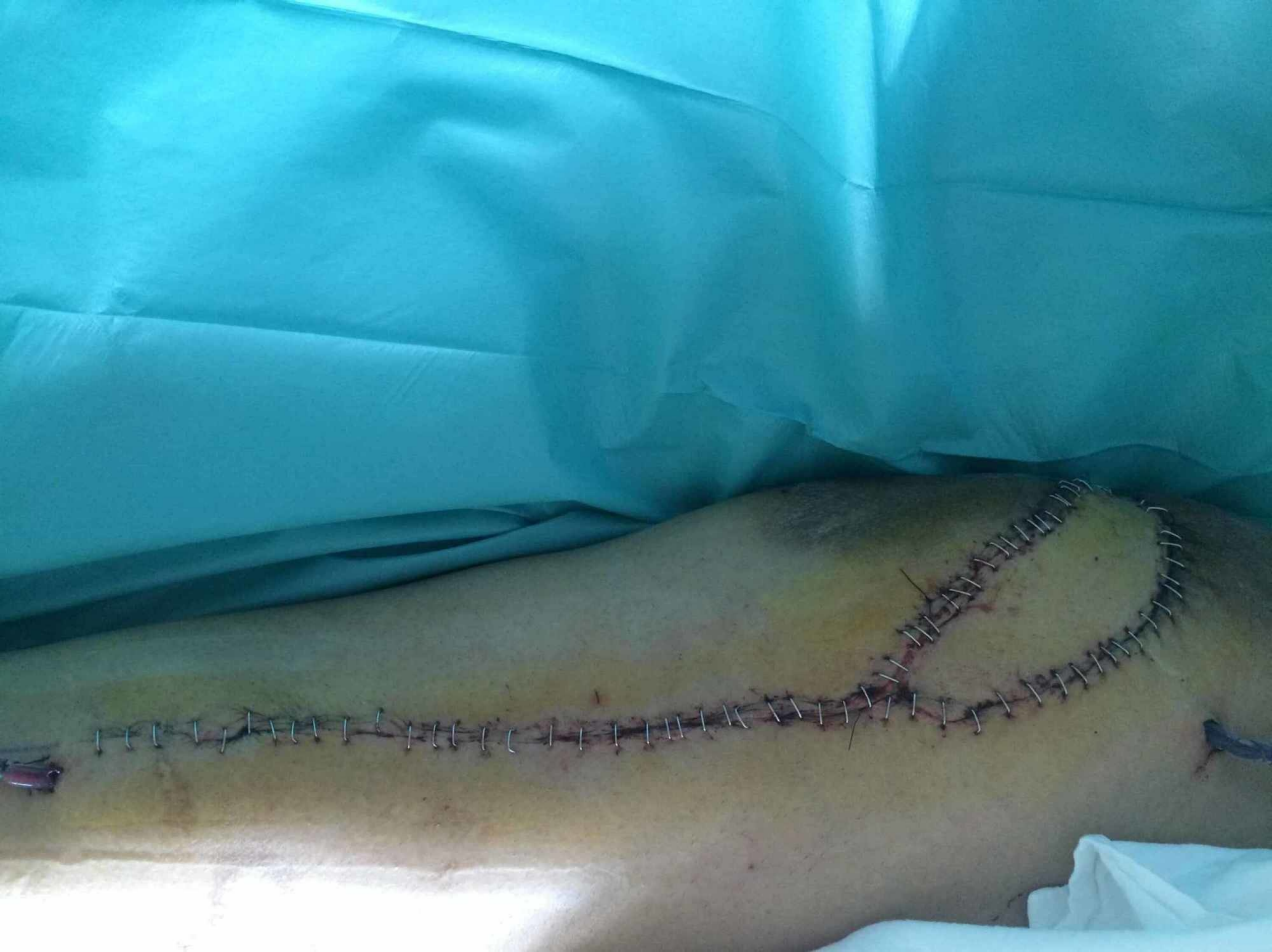
Reconstruction with rectus femoris plus vastus lateralis musculocutaneous flap.

The surgical procedure started by drawing a line on the thigh between the anterior superior iliac spine and the lateral border of the patella; an incision was made on the intermuscular septum between the rectus femoris and the vastus lateralis. The rectus was exposed and dissected; the distal insertion was cut, and the muscle was elevated proximally until the pedicle was visualized. Through the same incision, it was possible to dissect the vastus lateralis and gracilis muscle flaps. If necessary, a vastus lateralis musculocutaneous flap can be harvested. The vastus lateralis was divided from both the vastus intermedius and the distal insertion and also the gracilis can be isolated and dissected from the distal insertion. It was not necessary to dissect the two vascular pedicles because the arc of rotation was sufficient to reach the defect. The rectus flap and the gracilis flaps were used to fill the deep surface of the wound and fixed around the residual femur. The vastus lateralis flap was used to cover the wound; its deep surface was fixed to the rectus flap (Figure 2). The muscle was then fixed to the wound edges, and the skin paddle, or a skin graft in the case of a muscle flap, used to cover the flap (Figure 3).

**Figure 4:**
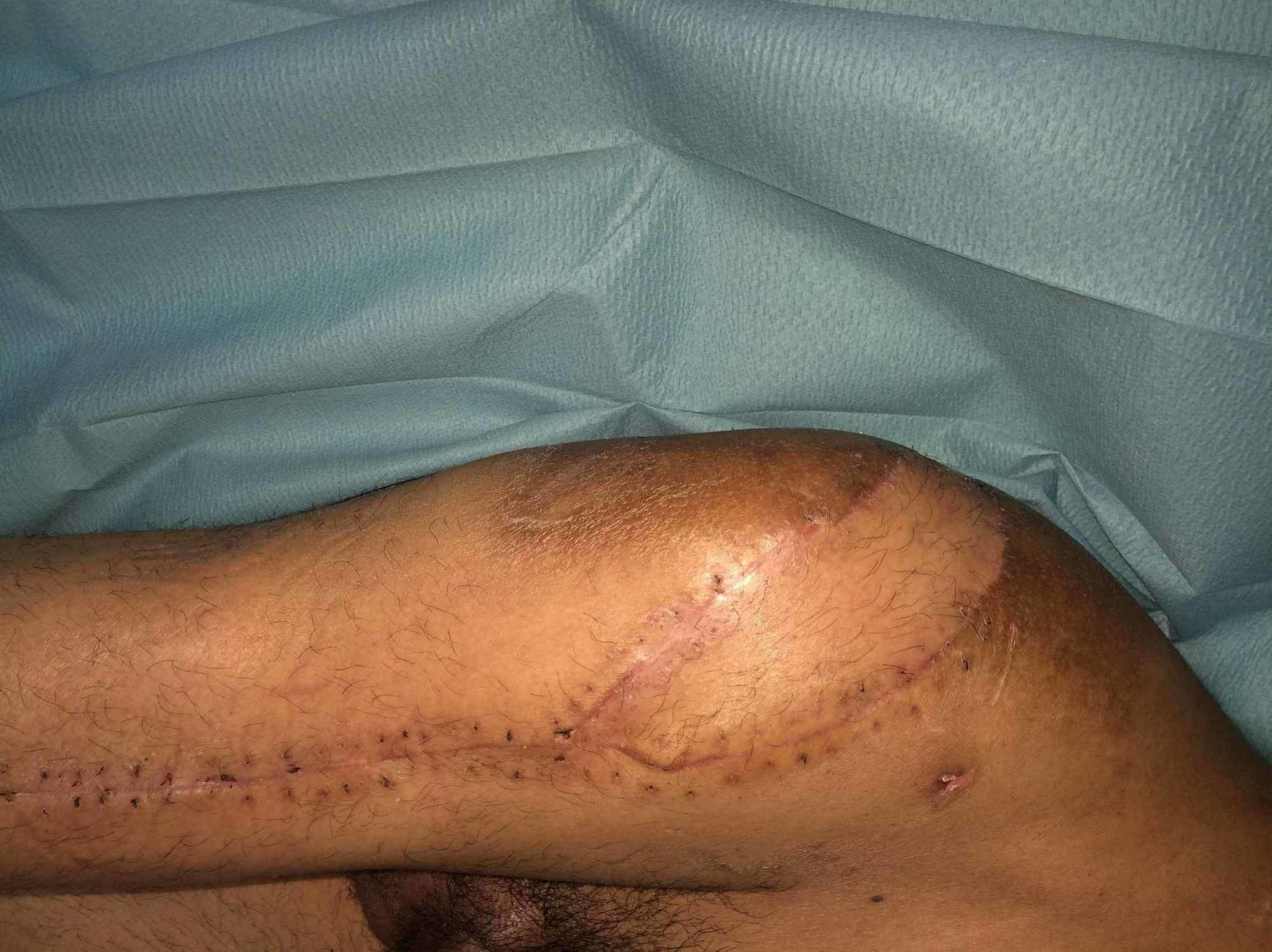
Result after 18 months.

Three drains were inserted and removed after one week, while sutures were removed after three weeks. During this period, the patient did not sleep on the wounds and did not sit on a chair. Antibiotic therapy was continued for eight weeks. After stitches were removed, we started having the patient sat for one hour per week and then increased this by one hour each week to four hours. Continued monitoring of the wounds was performed; no complications occurred in all the cases. After an average of 14 months (range, 10 to 18 months) of follow-up, the trochanteric region was completely healed with no signs of recurrence (Figure 4).

## Discussion

Trochanteric pressure sores are difficult to treat because they are frequently complicated by osteomyelitis involving the great trochanter and often extend to the femoris. In these cases, a wide debridement must be performed as the residual defect is large and deep. To obtain a good reconstruction, it is necessary to fill dead spaces and cover wound surface with adequate tissue to ensure healing and to reduce the risk of recurrence. In the case of infected wounds, we prefer to perform a surgical approach in two steps. In the first step, we perform the debridement and position a vacuum therapy, and in the second one, the reconstruction. Vacuum therapy allows us to reduce the size of the wound and the dead spaces while managing seromas. In the reconstructive procedure, the flap choice is guided by the amount of dead space to fill, so local muscle or musculocutaneous flaps are preferred [9]. The procedure also allows for the reconstruction of the defect with a good volume to ensure a reduction in the risk of recurrence. In spinal cord-injured patients, the leg muscles are generally atrophic, and a single flap may not provide enough bulk. The presence of dead spaces can lead to complications such as seroma and hematoma. Rectus femoris, vastus lateralis, and gracilis are muscles with nearby pedicle, easy to dissect, and can also be harvested as musculocutaneous flap using a large skin paddle. The dissection of each flap is made through a single incision on the thigh, and the donor site is easily closed by direct suture. The arc of rotation allows the flap to reach the deep surface of the defect with no tension, and the combination of a muscle flap positioned deeply and covered with a musculocutaneous flap permits full coverage of the defect and its surface with a skin paddle, or with a skin graft in case of a muscle flap. This approach is easy and can be applied in all cases of trochanteric sores. In our experience, free flaps are not needed because of many local flap availabilities. On the other hand, in case of recurrence after multiple local flaps rotation, a free flap from the contralateral lower limb might be considered. In SCI patients, we always avoid to harvest free muscle flaps from the trunk not to reduce muscular function.

## Conclusions

Trochanteric pressure sores are quite difficult to treat because they frequently result in large and deep wounds. The reconstruction must ensure a complete filling of all dead spaces and an adequate cover to reduce the risk of recurrence. In these cases, we believe that a two-step surgical approach can be useful. During the first step, debridement is performed to remove all unviable tissues. Then, antibiotic treatment permits the treatment of infection so that the reconstruction can be performed on a clean wound. In these cases, muscle or musculocutaneous flaps are the best choices because they permit the use of a good volume of viable tissue. In some cases, this kind of flaps can be combined to obtain a better result.
